# Reversibility of brain glucose kinetics in type 2 diabetes mellitus

**DOI:** 10.1007/s00125-022-05664-y

**Published:** 2022-03-05

**Authors:** Elizabeth Sanchez-Rangel, Felona Gunawan, Lihong Jiang, Mary Savoye, Feng Dai, Anastasia Coppoli, Douglas L. Rothman, Graeme F. Mason, Janice Jin Hwang

**Affiliations:** 1 Department of Internal Medicine/Section of Endocrinology, Yale University School of Medicine, New Haven, CT USA; 2 Department of Radiology and Biomedical Imaging, Yale University School of Medicine, New Haven, CT USA; 3 Department of Pediatric Endocrinology and General Clinical Research Center, Yale University School of Medicine, New Haven, CT USA; 4 Yale Center for Analytical Sciences, Yale School of Public Health, New Haven, CT USA; 5 Department of Biomedical Engineering, Yale University School of Medicine, New Haven, CT USA; 6 Department of Psychiatry, Yale University School of Medicine, New Haven, CT USA

**Keywords:** Brain glucose transport, Diabetes, Glucose kinetics

## Abstract

**Aims/hypothesis:**

We have previously shown that individuals with uncontrolled type 2 diabetes have a blunted rise in brain glucose levels measured by ^1^H magnetic resonance spectroscopy. Here, we investigate whether reductions in HbA_1c_ normalise intracerebral glucose levels.

**Methods:**

Eight individuals (two men, six women) with poorly controlled type 2 diabetes and mean ± SD age 44.8 ± 8.3 years, BMI 31.4 ± 6.1 kg/m^2^ and HbA_1c_ 84.1 ± 16.2 mmol/mol (9.8 ± 1.4%) underwent ^1^H MRS scanning at 4 Tesla during a hyperglycaemic clamp (~12.21 mmol/l) to measure changes in cerebral glucose at baseline and after a 12 week intervention that improved glycaemic control through the use of continuous glucose monitoring, diabetes regimen intensification and frequent visits to an endocrinologist and nutritionist.

**Results:**

Following the intervention, mean ± SD HbA_1c_ decreased by 24.3 ± 15.3 mmol/mol (2.1 ± 1.5%) (*p*=0.006), with minimal weight changes (*p*=0.242). Using a linear mixed-effects regression model to compare glucose time courses during the clamp pre and post intervention, the pre-intervention brain glucose level during the hyperglycaemic clamp was significantly lower than the post-intervention brain glucose (*p*<0.001) despite plasma glucose levels during the hyperglycaemic clamp being similar (*p*=0.266). Furthermore, the increases in brain glucose were correlated with the magnitude of improvement in HbA_1c_ (*r* = 0.71, *p*=0.048).

**Conclusion/interpretation:**

These findings highlight the potential reversibility of cerebral glucose transport capacity and metabolism that can occur in individuals with type 2 diabetes following improvement of glycaemic control.

**Trial registration** ClinicalTrials.gov NCT03469492.

**Graphical abstract:**

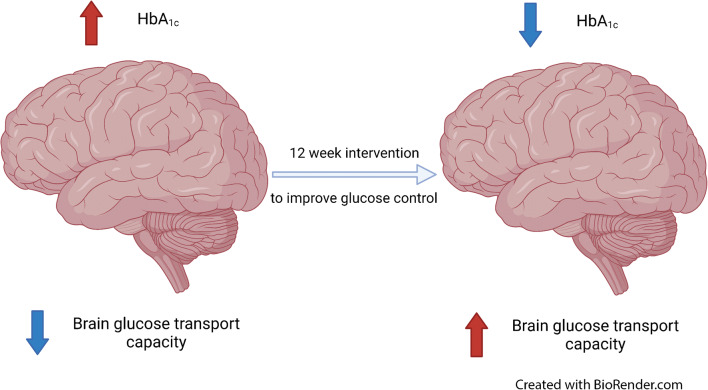

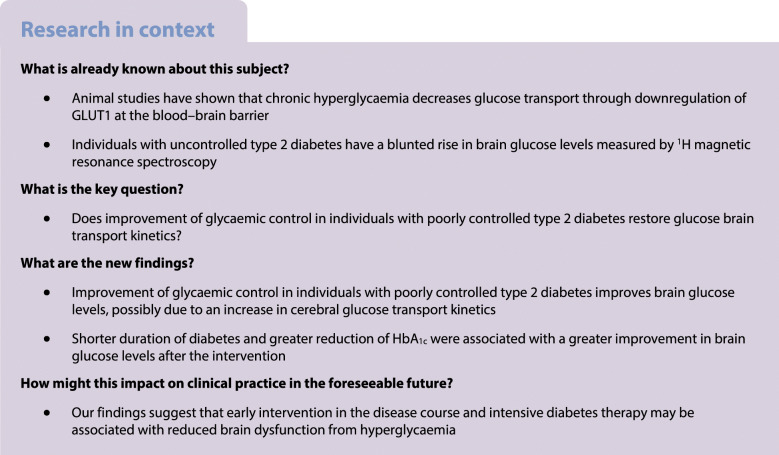



## Introduction

The prevalence of type 2 diabetes mellitus in the USA and worldwide continues to rise and despite many available treatment options many diabetic individuals fail to achieve glycaemic targets leading to increased morbidity and mortality [1, 2]. Cognitive impairment [3, 4] and dementia [5] have been recognised as clinically important complications arising from chronic exposure to hyperglycaemia. Furthermore, chronic hyperglycaemia has been shown to lead to abnormalities of cerebral cellular metabolic processes as well as changes in GLUT function and expression [6–8].

Glucose, the main energy source of the brain, is supplied across the blood–brain barrier (BBB) via facilitated diffusion through glucose transporters, principally GLUT1 [9]. Studies in healthy, lean individuals using magnetic resonance spectroscopy (MRS), a non-invasive imaging technique that measures concentrations of metabolites in living tissue, have shown that brain glucose levels rise in a linear fashion with rising plasma glucose levels [10]. In animals, chronic hyperglycaemia decreases glucose transport through downregulation of GLUT1 at the BBB [11]. In previously published work, our group showed that compared with lean participants without diabetes, brain glucose increments in response to a standardised increase in plasma glucose levels were lower in participants with poorly controlled type 2 diabetes [12], suggesting that chronic hyperglycaemia is associated with blunted brain glucose transport and/or metabolism. Whether these changes in cerebral glucose transport/metabolism seen in individuals with poorly controlled diabetes can be reversed is unknown. This proof of concept study was therefore undertaken to determine whether improvement of glycaemic control in individuals with poorly controlled type 2 diabetes would restore glucose brain transport kinetics.

## Methods

### Study design and participants

This is a pre–post-intervention trial to measure changes in cerebral glucose levels at baseline and after a 12 week intervention to improved glycaemic control through the use of continuous glucose monitoring (CGM), diabetes regimen intensification and frequent visits to an endocrinologist and nutritionist.

Eight individuals with poorly controlled type 2 diabetes defined as a HbA_1c_ ≥58 mmol/mol (7.5%), aged 18–65 years, and BMI ≥18 km/m^2^, were recruited from the greater New Haven between January 2018 and November 2019 and participated in the study (Table 1). Participants provided informed consent and were on a stable regimen of insulin or oral diabetes medications for at least 90 days before the first scan. Participants 1 and 4 were treated with basal/bolus insulin regimen. Participant 2 used basal insulin, metformin, sodium–glucose cotransporter 2 (SGLT2) inhibitor and dipeptidyl peptidase 4 (DPP-4) inhibitor. Participant 3 was treated with basal/bolus insulin, metformin and an SGLT2 inhibitor. Participant 5 was using basal insulin, metformin and a DPP-4 inhibitor. Participant 6 was using basal insulin, metformin and a sulfonylurea. Participant 7 was using metformin, and participant 8 was using a sulfonylurea only. Individuals were excluded if they had type 1 diabetes, known psychiatric or neurological disorders, active infection, malignancy, abnormal thyroid function, cerebrovascular disease, cardiovascular disease, hepatobiliary disease, or significant weight change in the last 3 months (more than 5% weight change). Other exclusion criteria included inability to enter the MRI scanner, illicit drug use and smoking history assessed by interview, use of medications that could have effects on the brain including psychiatric medications, or recent steroid use. Women who were breastfeeding, seeking pregnancy or had a positive urine pregnancy test were also excluded.

**Table 1:** Participant characteristics at baseline (week 0) and after intervention (week 12)

Variable	Week 0	Week 12	*p* value
*N*	8		
Sex, male/female, *n* (%)	2 (25) /6 (75)		
Age, years	44.8 ± 8.3		
Duration of diabetes, years	10.0 ± 8.2		
Weight, kg	85.0 ± 21.2	83.2 ± 20.0	0.242
BMI, kg/m^2^	31.4 ± 6.1	30.6 ± 5.4	0.085
HbA_1c_, mmol/mol	84.1 ± 16.2	59.7 ± 10.1	0.006
HbA_1c_, %	9.8 ± 1.4	7.7 ± 0.9	0.006

Data are expressed as mean ± SD or *n* (%)

Paired *t* tests were used to determine differences within groups

### Experimental protocol

#### Brain glucose measurements using ^1^H MRS scanning

Participants underwent ^1^H MRS scanning at 4 Tesla during a hyperglycaemic clamp at baseline and after a 12 week intervention to improve glycaemic control. On the evening prior to each MRS scan, individuals were admitted to the Yale University Hospital Research Unit and placed on a standard hospital protocol insulin drip overnight to normalise plasma glucose levels. The insulin infusion was continued as needed to maintain plasma glucose at 5 mmol/l until the start of the hyperglycaemic clamp.

For the hyperglycaemic clamp, a variable 20% dextrose infusion was adjusted every 5–10 min for 2 h so that plasma glucose levels were increased from baseline to the target level of 12.2 mmol/l as previously described [12]. Venous blood was drawn to sample insulin levels every 2 min for the first 10 min and every 30 min thereafter.

**Fig. 1:**
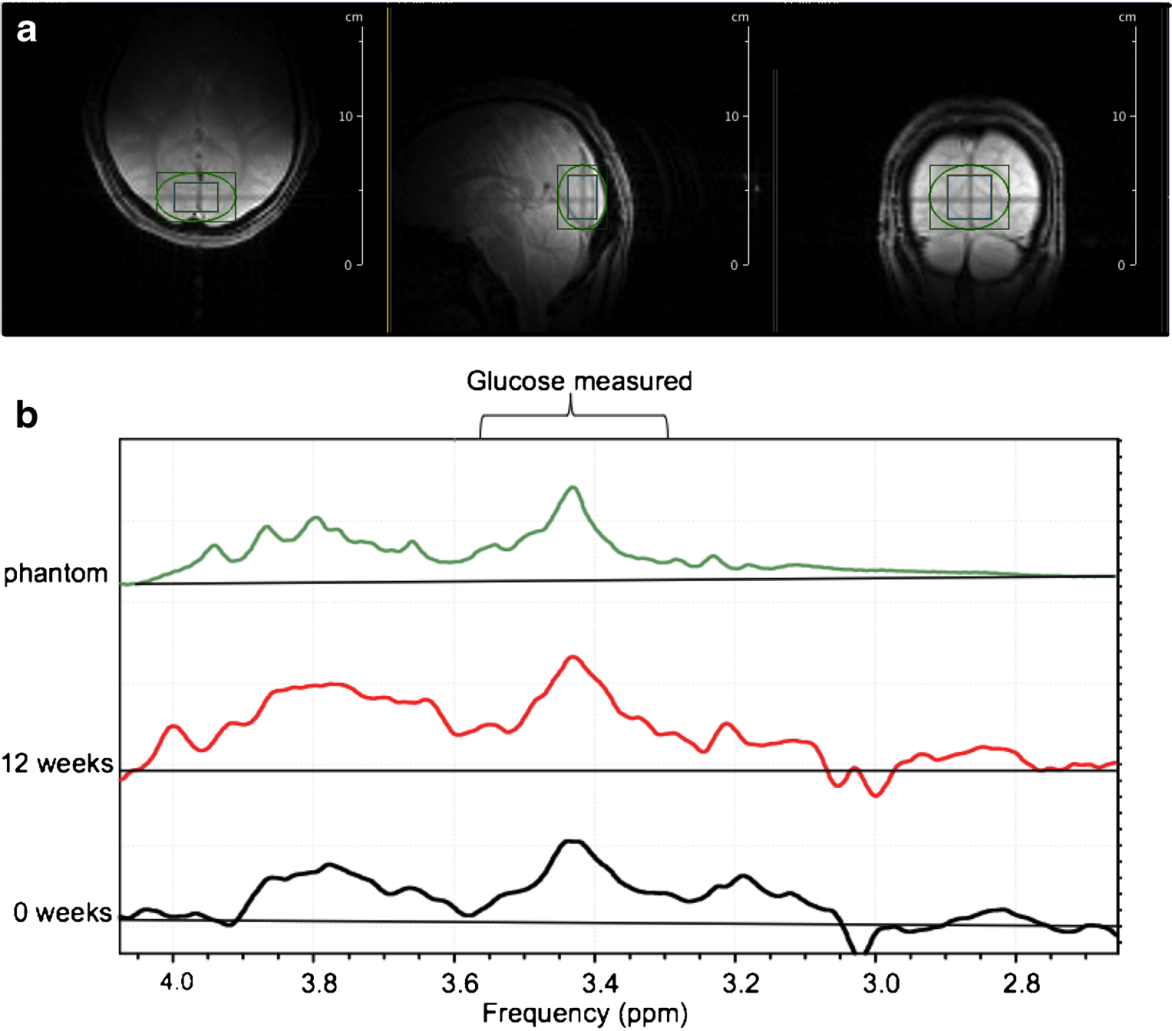
^1^H scanning of the brain. (**a**) Voxel placement, a 30 × 20 × 30 mm voxel was centred at the midline of the occiput. Localised shimming was obtained from an elliptical volume (green) around the selected voxel. The scale on the image is in (cm) centimeters (vertical axis). (**b**) Representative difference spectra at baseline (week 0, black) and after the intervention (week 12, red) from 1 participant. The glucose reference spectrum (phantom) is shown in green. The spectral window in which the glucose integration was performed is indicated

For ^1^H MRS scanning, participants were positioned supine in a 4.0 T whole-body magnet interfaced to a Bruker ParaVision 6.0 spectrometer (Bruker Instruments, USA), with the head immobilised using foam inserts to lie within a quadrature surface radiofrequency probe with two orthogonal circular surface coil transceivers as previously described [13, 14]. After tuning, calibration and acquisition of scout images for anatomical localisation, intracerebral glucose concentration signals were obtained using stimulated echo acquisition mode (STEAM) localisation [15] in a 30 × 20 × 30 mm^3^ voxel in the occipital lobe. To ensure reproducible positioning, the volume was centred at the midline of the occiput with the volume centre along the *z*-axis set 15 mm above the cerebellum and the volume centre along the *y*-axis set 10 mm above the surface of the occipital cortex at the midline (see Fig. 1).

Spectra were obtained for two 10 min blocks of baseline then every 10 min for at least 2 h of hyperglycaemia.

Shimming was performed by obtaining B_0_ mapping, followed by iterative local shimming, using an elliptical shim volume centred on the voxel (Fig. 1). The standard Bruker STEAM [16] sequence was used with repetition time (TR) = 2000 ms, echo time/mixing time (TE/TM) = 15 ms/10 ms, bandwidth = 5000 Hz and sampling points = 2048. Each 10 min spectrum was the average of 296 scans, with VAriable Power and Optimized Relaxations delays (VAPOR) for water suppression [17]. Spectra were acquired with a B_0_-lock during the interscan delay to compensate for field drift using a navigator pulse (Gauss, 0.548 ms, bandwidth 5000 Hz, flip angle 10^o^).

#### Spectral analysis

**Fig. 2:**
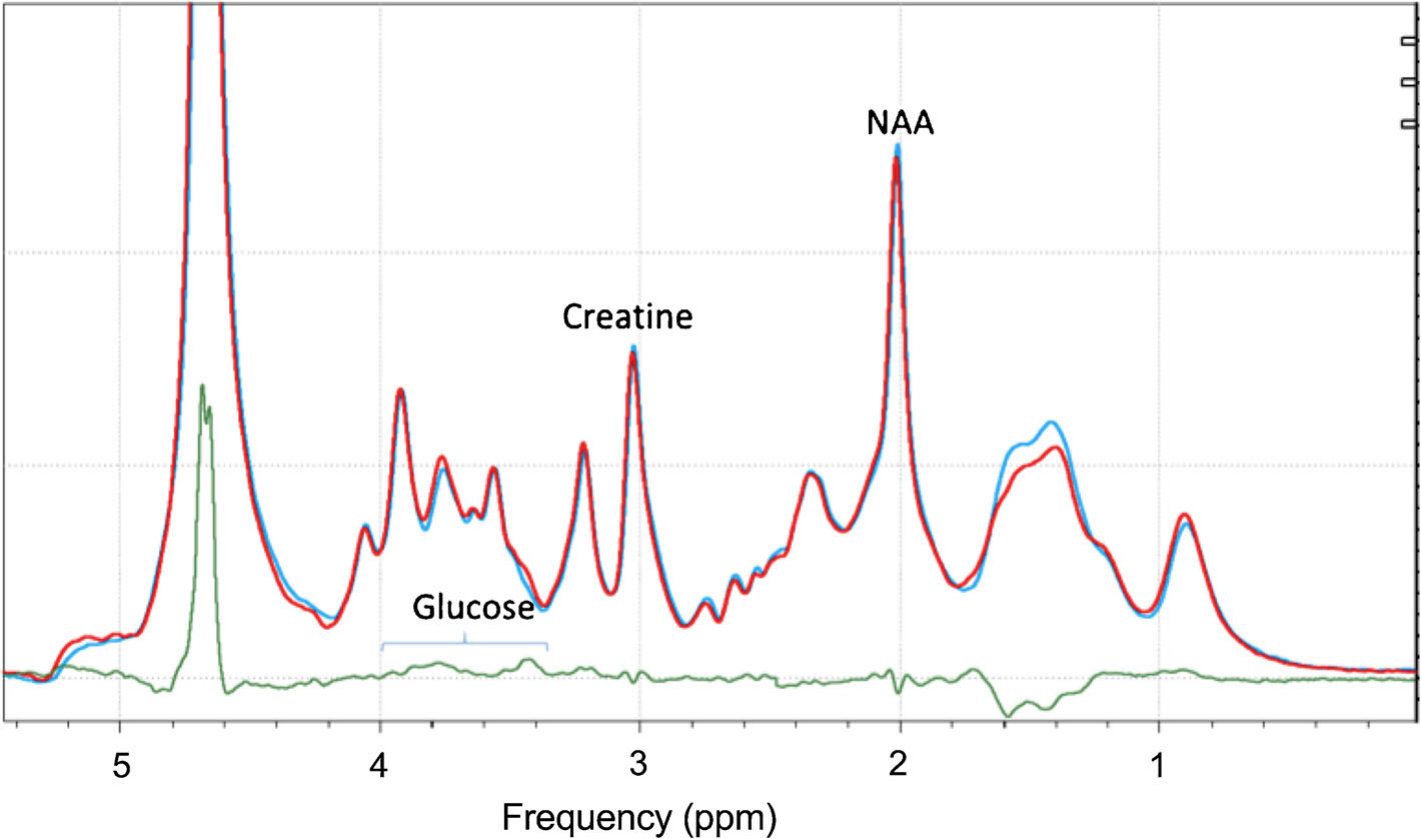
Spectral processing for intracerebral glucose. An example using one representative participant of how an individual time point is calculated for change in brain glucose levels using difference spectra. The blue spectrum was obtained at baseline and the red spectrum was obtained 10 min later. The green spectrum underneath is the difference between the red and blue spectra

Fourier transformation was performed using an exponential apodisation with a Lorentzian half width of 4 Hz. The sum of the baseline spectra was subtracted from each spectrum obtained during the glucose infusion to eliminate overlap from other brain metabolites as previously described [18, 19]. The differences between spectra were done with matching line width, phasing and frequency shifting, without adjustment of intensity (Fig. 2). Changes in glucose levels were measured by peak integration of the difference spectrum, integrating the glucose C_3_H-C_5_H peak from 3.32 to 3.54 ppm [20]. Baseline corrections were performed in the range of 0 ppm to 4.0 ppm. This partial glucose integral was converted to full intensity using a correction factor. The correction factor was obtained by acquiring spectra of free glucose in a solution using the same pulse sequence and then determining the ratio between the integral from 3.32 and 3.54 ppm with the integral obtained between 3.10 and 3.96 ppm (which contains the ^1^H MRS resonances of C2 through C6 glucose). The free glucose spectrum was line broadened to approximate the linewidths seen in vivo. For conversion to concentration, the corrected partial glucose integral was divided by the integral of the resonance of the CH_3_ group of creatine between 2.83 and 3.11 ppm in the baseline spectrum, multiplied by the correction factor, and corrected for the number of protons contributing to the resonances. The creatine concentration was assumed to be 10 mmol/kg tissue, as previously described [12]. The spectral processing described above was performed using the Bruker Topspin 3.2 software (https://www.bruker.com/en/products-and-solutions/mr/nmr-software/topspin.html). Most spectra were acquired with a water linewidth of 8.5–11 Hz.

To rule out the possibility that the changes in the increase in glucose levels observed were due to spectroscopic variation, we also determined the choline, creatine, and *N*-acetylaspartate (NAA) concentrations for both scans of all participants using LCModel [16] obtained at the beginning of each scanning session prior to glucose infusion. In all cases, the variation between scans was less than 10%, which is well below the changes in glucose measured. The per cent change in creatine/water ratio before and after the intervention was calculated to be 0.62%, indicating the high stability of the measurement and consistent MRS volume location since creatine concentration is lower in white matter [21]. Additional evidence of consistent volume location was that the NAA/creating ratio, which also depends upon grey/white matter composition, did not change.

### Intervention protocol

#### CGM

Prior to the first MRS scanning session, participants were asked to wear a blinded continuous glucose monitor (Freestyle Libre Pro; Abbott, USA). For calculation of metrics of glycaemic variability, the values obtained were imported into Easy Glucose Variability (EasyGV) software version 9.0.R2 (www.phc.ox.ac.uk/research/technology-outputs/easygv) [22] using a method adapted from Chan et al [23] and used in a prior study [24].

Following the first MRS scan, and for the duration of the 12 week intervention, participants were provided with personal unblinded Freestyle Libre CGM (FSL-CGM) to allow for self-monitoring of their glucose control. Participants met with a research team member every 1–2 week(s) to review data in person or over the phone as described below. All participants received instructions and training on the proper use of the CGM system.

#### Diabetes medication management

Participants met regularly with an endocrinologist to review glucose measurements from the FSL-CGM system every 1–2 week(s). Insulin doses and oral glucose-lowering drugs were adjusted following ADA recommendations for fasting and postprandial glucose goals. Awareness of blood glucose change during exercise was emphasised and treatment of hypoglycaemia was reviewed.

#### Nutrition/lifestyle visit

All participants met regularly with a registered dietitian (RD), who was also a certified diabetes educator (CDE), to review diet and physical activity habits. The initial visit focused on reviewing typical food intake and setting goals to ensure that the diet was nutrient-dense and that the participant was aware of carbohydrate intake and its effect on blood glucose. The RD was sensitive to each individual’s nutritional needs based on personal and cultural preferences. Physical activity level was also assessed and goals were individualised, as was done for nutritional recommendations. If a participant was engaging in minimal activity, a gradual increase was recommended at each visit. Those already engaging in regular activity of moderate-to-vigorous intensity and flexibility training were encouraged to continue with their regimen. Overall, participants were advised to decrease sedentary activity such as watching TV and internet surfing. All diet and exercise counselling was in accordance with the ADA 2018 guidelines [25]. Participants met with the RD on weeks 1, 3, 5, 8 and 11 and weight was recorded at each visit. So that weight change would not confound study outcomes, interventions were tailored to keep participants’ weight stable (±5 lb [2.23 kg]).

#### Laboratory analysis

Plasma glucose levels were measured via glucose oxidase (YSI; Xylem, USA). Plasma insulin levels were measured by double-antibody RIA (Millipore, Merck, USA).

### Statistics

Analyses of every repeatedly measured outcome, including intracerebral glucose and plasma glucose levels, were performed using the linear mixed-effects regression model method as previously described [12, 24, 26]. The pre-intervention (week 0) and post-intervention (week 12) brain glucose measures from the same participants were first aligned together by the same measurement time points (every 5–10 min during the hyperglycaemic clamp) and then transposed by week into a dataset of long format, in which a pair of pre and post measures per time point was saved in a single column for each participant. The SAS Proc MIXED procedure was used to fit the model, with three independent variables including a categorical time variable, a dummy week variable (week 0 and week 12), and the time by week interaction term. An unstructured covariance matrix was specified to account for the within-correlation between weekly paired values (week 0 and week 12), with an extra random subject effect introduced to account for the correlation of the minute-level measures during the hyperglycaemic clamp within a participant. In a separate analysis, glucose levels obtained every 5–10 min between times 60–120 min of the clamp were averaged to calculate a mean steady-state glucose level. Paired *t* tests were then used to compare these steady-state mean values before and after the intervention. Data analyses were performed using SAS, version 9.4 (Cary, USA) and SPSS software (version 26.0; IBM, USA). *p* values of less than 0.05 were considered statistically significant. No adjustment for multiple comparisons was considered for the exploratory endpoints in the current study.

### Study approval

The Yale University Human Investigation Committee approved the protocol, and all participants provided written informed consent prior to study participation.

## Results

### Participant characteristics

As shown in Table 1, two male and six female participants had a mean ± SD age of 44.8 ± 8.3 years and had a mean duration of diabetes of 10.0 ± 8.2 years. They had poorly controlled diabetes, as demonstrated by a mean ± SD HbA_1c_ of 84.1 ± 16.2 mmol/mol (9.8 ± 1.4%) at baseline. HbA_1c_ was significantly reduced after the intervention with an average decrease of 24.3 ± 15.3 mmol/mol (2.1 ± 1.5%) (*p*=0.006). There were no significant changes in weight (mean ± SD weight was 85.0 ± 21.2 kg before the intervention and 83.2 ± 20.0 kg after the intervention, *p*=0.242).

### Plasma and brain glucose levels

**Fig. 3:**
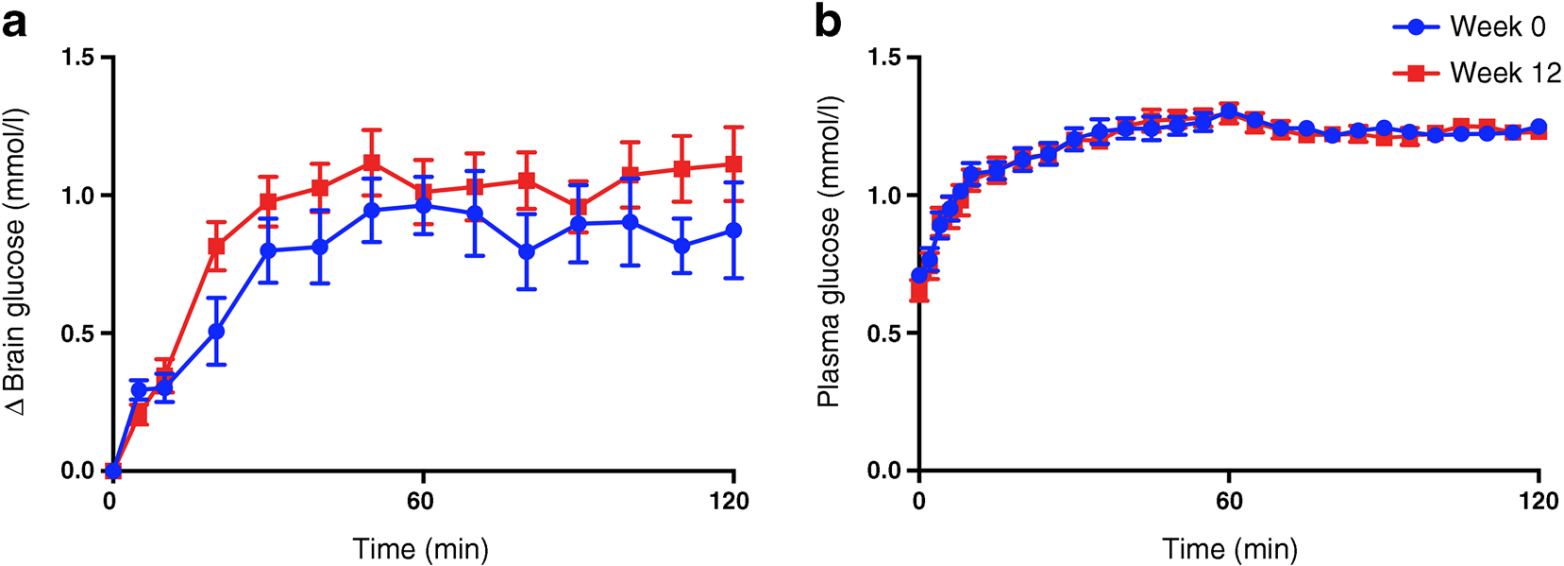
Change in intracerebral glucose and plasma glucose levels. (**a**) Mean change in intracerebral glucose concentrations over time at week 0 and 12. (**b**) Mean plasma glucose levels over time. All eight participants were included in analysis. Data are presented as mean ± SEM

Using mixed-effects regression models, which take into account the repeated measures of brain and plasma glucose over the hyperglycaemic clamp, the pre-intervention brain glucose level during the hyperglycaemic clamp was significantly lower than the post-intervention brain glucose (*p*<0.001; Fig. 3a) despite plasma glucose levels during the hyperglycaemic clamp being similar (*p*=0.266; Fig. 3b). No significant differences in fasting plasma insulin levels were observed (mean ± SD: 62 ± 68 μU/ml in week 0; 31 ± 22 μU/ml in week 12; *p*=0.20). Similarly, there were no differences in mean plasma insulin levels measured during time 60–120 min of the hyperglycaemic clamp (mean ± SD: 31 ± 26 μU/ml in week 0; 38 ± 29 μU/ml in week 12; *p*=0.25). Mean ± SD glucose infusion rates (GIRs) during the hyperglycaemic clamps were 1.61 ± 1.09 mg kg^−1^ min^−1^ in week 0 and 2.75 ± 1.61 mg kg^−1^ min^−1^ in week 12 (*p*=0.07). Correlation between change in GIR and change in brain glucose measures was not significant (*r* = 0.3, *p*=0.45).

There were also no differences in pre- vs post-intervention concentrations of other intracerebral metabolites, including NAA, glutamine, glutamate, glycerophosphocholine + choline and myoinositol, at baseline vs after the intervention (Table 2).

**Table 2:** ^1^H MRS metabolites at baseline and after intervention

Metabolite	Week 0	Week 12	*p* value
NAA + NAAG	1.60 ± 0.15	1.51 ± 0.82	0.10
Creatine + PCr	1.00	1.00	–
Glutamine	0.28 ± 0.07	0.30 ± 0.06	0.38
Glutamate	1.07 ± 0.07	1.04 ± 0.08	0.40
Glu + Gln	1.35 ± 0.11	1.35 ± 0.13	0.99
GPC + Choline	0.21 ± 0.02	0.21 ± 0.02	0.98
Myoinositol	0.62 ± 0.05	0.60 ± 0.04	0.15

Data are expressed as mean ± SD of concentrations relative to (Creatine+PCr) × 10

PCr, phosphocreatine; GPC, glycerophosphocholine

### Reduction in HbA_1c_ and change in brain glucose levels

**Fig. 4:**
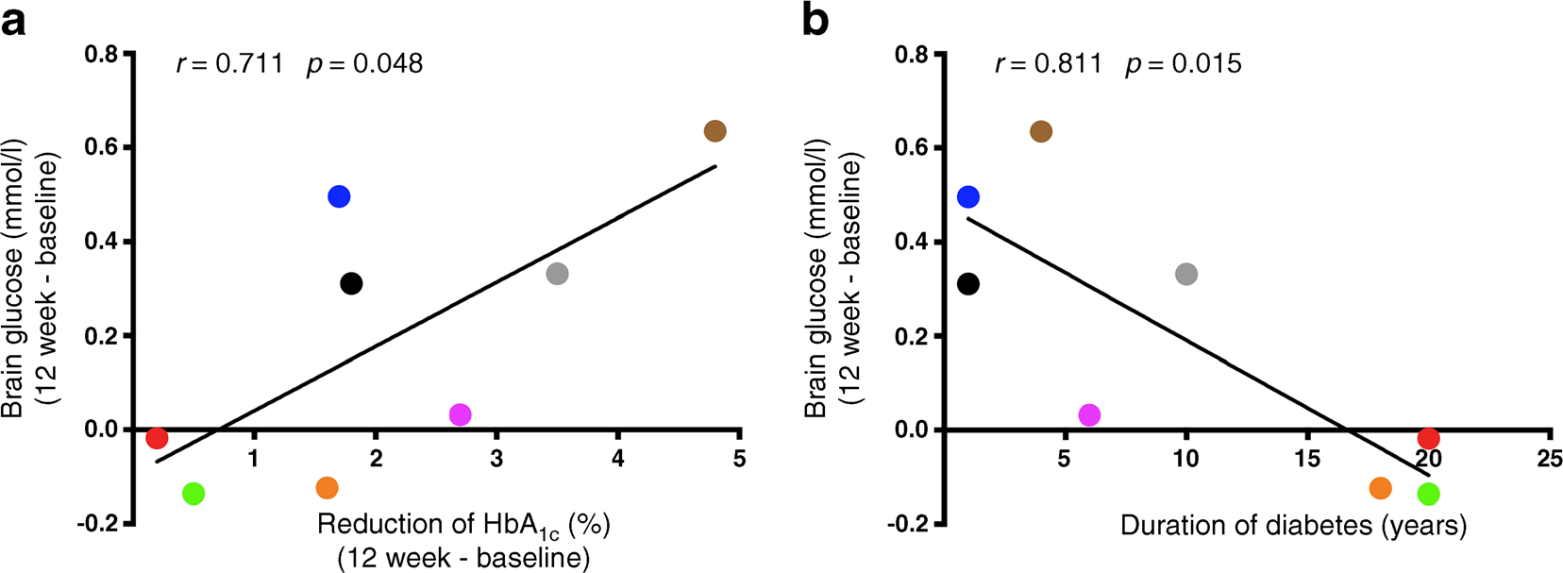
Relationship between change in intracerebral glucose levels, reduction in HbA_1c_ and duration of diabetes. All eight participants were included in the analysis. (**a**) Change in the average of brain glucose levels at steady state (time 60–120 min) correlated using a Pearson’s correlation with reduction of HbA_1c_ (12 weeks vs 0 weeks). (**b**) Change in the average of brain glucose levels at steady state (time 60–120 min) correlated using a Pearson’s correlation with duration of diabetes

To assess factors that might be driving these changes, we examined the relationship between the mean change in brain glucose levels at steady state (averaged between time 60–120 min) and reductions in HbA_1c_. The mean brain glucose level (time 60–120 min) was 1.07 ± 0.23 mmol/l before the intervention and 0.88 ± 0.35 mmol/l after the intervention (*p*=0.10). There was a significant correlation between the change in brain glucose levels and the degree of reduction in HbA_1c_. Individuals who displayed greater reduction in HbA_1c_ after the intervention had greater increases in brain glucose (*r* = 0.711, *p*=0.048) (Fig. 4a).

### Duration of diabetes and change in brain glucose levels

There was a significant correlation between the mean change in brain glucose levels at steady state (time 60–120 min) before and after the intervention and the duration of diabetes. As seen in Fig. 4b, shorter duration of diabetes was correlated with a greater change in brain glucose levels after the intervention (*r* = −0.811, *p*=0.015). Correlations between duration of diabetes and change in HbA_1c_ during the intervention (*r* = −0.582, *p*=0.131) and age of participants (*r* = −0.26, *p*=0.526) were not significant.

### CGM data

As seen in Table 3, based on the CGM reports obtained prior to each scanning session, the percentage of time that participants spent in mild hypoglycaemia (glucose concentration <3.89 mmol/l) and in moderate hypoglycaemia (glucose concentration <2.77 mmol/l) did not differ between week 0 and week 12.

**Table 3:** CGM data at baseline and after intervention

CGM data	Week 0	Week 12	*p* value
% Time, glucose <3.89 mmol/l	0.7 ± 2.1	0.6 ± 1.3	0.666
% Time, glucose <2.77 mmol/l	0.4 ± 1.0	0.0 ± 0	0.351
% Time, glucose >9.99 mmol/l	69.3 ± 32.6	32.5 ± 17.0	0.034
% Time, glucose ≥3.89 mmol/l to ≤9.99 mmol/l	29.5 ± 31.4	66.8 ± 17.1	0.030
No. of episodes, glucose <2.77 mmol/l	1.3 ± 3.8	0.0 ± 0	0.351
No. of episodes, glucose <3.89 mmol/l	3.3 ± 9.5	2.8 ± 6.1	0.705
EasyGV data^a^			
Mean	13.0 ± 4.3	8.8 ± 1.5	0.047
SD	3.3 ± 0.7	2.4 ± 0.9	0.082
CONGA	10.8 ± 4.0	7.0 ± 1.0	0.049
LI	14.3 ± 6.4	10.0 ± 7.7	0.084
JINDEX	94.1 ± 52.8	43.0 ± 15.2	0.040
LBGI	1.3 ± 2.2	1.7 ± 0.9	0.620
HBGI	22.5 ± 14.5	7.8 ± 3.9	0.031
GRADE	16.2 ± 9.9	7.2 ± 3.3	0.048
MAGE	7.8 ± 1.6	5.9 ± 2.4	0.126
*M* value	42.1 ± 37.5	8.0 ± 4.6	0.037
MAG	5.8 ± 1.2	5.0 ± 1.7	0.161

Data are presented as mean ± SD

CGM variables were calculated from the 5 days before MRS scanning

^a^Different glucose variability measures were calculated from the 5 days prior to the MRS scanning as per [22] using EasyGV software

CONGA, continuous overlapping net glycaemic action; GRADE, glycaemic risk assessment in diabetes equation; HBGI, high blood glucose index; JINDEX, index of glycaemic control; LBGI, low blood glucose index; LI, lability index; *M* value, Morbus value; MAG, mean absolute glucose; MAGE, mean amplitude of glucose excursions

The percentage of time spent in hyperglycaemia (glucose concentration >9.99 mmol/l) decreased after the intervention (*p*=0.034). No significant associations were observed for rates of mild hypoglycaemia or moderate hypoglycaemia, or percentage of time spent in hypoglycaemia or hyperglycaemia, and brain glucose levels. Furthermore, using the software program EasyGV [22], we calculated common metrics of glycaemic variability from the CGM and no significant correlations were observed between metrics of glycaemic variability and brain glucose measurements either at baseline or after the intervention.

## Discussion

In this proof of concept study, we show that improved glycaemic control results in a 20% increase in brain glucose levels. Furthermore, the degree of this increase correlated with the magnitude of HbA_1c_ improvement. These findings suggest that human brain glucose transport and/or metabolism may be restored by good glycaemic control.

The relationship between brain glucose levels and circulating plasma glucose levels based on human and animal model studies has been shown to be linear [10, 27]. We have shown that the slope of this linear relationship, which we determine in our glucose difference measurement, can be used to calculate the ratio of the maximum transport activity (Tmax) to the rate of glucose metabolism (CMRglc) [12]. How glucose transport activity and metabolism and, as a result, brain glucose levels, are altered in response to hypo- or hyperglycaemia has been the subject of study for decades. Prior work by other groups using positron emission tomography (PET) [28], arterio-venous difference studies [29] and ^13^C MRS studies [30] have shown minimal differences in CMRglc when between individuals with poorly controlled type 1 diabetes and age-matched healthy control individuals under euglycaemic conditions. Furthermore, rodent studies have found that diabetes is associated with cerebral hypometabolism [31]. Taken together with our current findings, this suggests that the greater increase in brain glucose concentrations seen after intervention could be due to improvements in GLUT activity/capacity.

Chronic exposure to hyperglycaemia has been shown in both animal and human studies to decrease glucose transport into the brain [12, 24, 32]. This has been postulated to be a protective adaptation of the central nervous system (CNS) against the deleterious consequences of hyperglycaemia [33, 34]. Similarly, upregulation of glucose transport into the brain following repeated exposure to hypoglycaemia is postulated to be an important adaptive mechanism to ensure adequate glucose supply to the brain in the setting of hypoglycaemia. This hypothesis is supported by animal studies showing upregulation of *Glut1* in rodents subjected to repeated hypoglycaemia [35, 36]. Likewise, human studies in individuals with type 1 diabetes have found that frequent hypoglycaemia may lead to increased glucose entry into the brain [29]. However, despite compelling evidence to support adaptive changes at the BBB to increase or decrease brain glucose transport, there have also been rigorous human studies that have not found differences in brain glucose in response to hyperglycaemia [19, 37] or hypoglycaemia exposure [38, 39]. These disparate conclusions have been attributed to differences in study designs, participant populations and neuroimaging methodologies, and thus it remains unclear exactly what factors in addition to circulating glucose levels can impact transport of glucose into the brain.

Furthermore, very few studies have investigated the reversibility of CNS metabolite changes in response to improvement in glycaemic control. To our knowledge, prior to our current study, there have been no other human studies investigating the reversibility of brain glucose transport/metabolism. Our study participants all had poorly controlled type 2 diabetes with minimal hypoglycaemia and modest levels of glycaemic variability. Because the 12 week intervention effectively lowered exposure to high glucose levels without increasing rates of variability or hypoglycaemia, we conclude that the improvement in brain glucose responses are predominantly due to decreased exposure to hyperglycaemia. Our findings are consistent with work by other groups using different neuroimaging modalities, such as functional MRI brain scanning, which found that better glycaemic control can modify brain function in the short term. For example, improvement of HbA_1c_ following gastric bypass surgery was associated with significant changes in regional brain activity patterns 6 months after surgery [40] and the extent of brain activity changes also correlated with the degree of improvement in HbA_1c_ [40].

In our present study, we also observed that the degree of change in brain glucose levels correlated with the magnitude of improvement in HbA_1c_ (Fig. 4a). In addition, individuals with shorter duration of diabetes also had a greater improvement in brain glucose levels (Fig. 4b). While these post hoc analyses must be interpreted cautiously given our small sample size, this suggests that early and prompt interventions to improve diabetes control may be associated with greater improvements in CNS complications of diabetes. This finding resonates with the conclusion of early studies of diabetes microvascular complications, highlighting the importance of early implementation of intensive glucose management [41, 42].

Improved sensitivity of the brain to the changes in circulating glucose levels could have implications for peripheral glucose homeostasis as well as eating behaviour. In animal studies, glucose sensing in the brain regulates peripheral glucose homeostasis [43–45]. In our study, we did not observe any relationships between brain glucose levels and surrogate measures of insulin sensitivity including GIR or insulin secretion. Future studies will be needed to clarify whether improvement in brain glucose could have any effects on peripheral measures of glucose homeostasis.

Finally, one of the primary mechanisms of neuronal damage from diabetes arises from hyperglycaemia-driven oxidative stress [34]. Diabetes is a well-established risk factor for cerebrovascular disease [46], dementia [3] and neurodegenerative diseases such as Alzheimer’s disease [47]. While it is without question that intensive glucose control will delay the onset and progression of microvascular and macrovascular complications of type 2 diabetes [48, 49], it is less clear whether tight glycaemic control can improve cognitive dysfunction. Many studies have failed to show that better glycaemic control can improve cognitive function among individuals with diabetes [50–52]. Further studies will be needed to investigate the impact of changing brain glucose transport kinetics on neurocognitive function.

The present study has several limitations. First, while participants were studied under carefully controlled hyperglycaemic clamp conditions with nearly identical plasma glucose levels, our brain glucose findings should be interpreted with caution due to the small sample size. Future, adequately powered studies with equal distribution of men and women will be needed to confirm our findings. Second, we did not have a control group and it is not possible to separate the intervention effects from potential time effects from participating in a clinical trial. In addition, our brain glucose measurements were obtained in the occipital cortex due to its relative ease of access and reliability for spectral acquisition. Glucose concentrations have been reported to be different between grey and white matter [10] and we did not specifically correct for grey/white matter in this study; however, we used a relatively large voxel size placed using anatomical landmarks to ensure that the voxel was placed in the same location for each participant before and after the intervention. In addition, there is evidence for regional differences in brain glucose metabolism [53] and further studies will be needed to measure brain glucose uptake in other regions of the brain such as the hypothalamus. Finally, because we did not use labelled glucose infusions (such as with [^13^C]glucose), we were not able to trace the metabolic fate of glucose or measure the cerebral metabolic rate of glucose.

In summary, our data show that in individuals with poorly controlled type 2 diabetes, improvement of glycaemic control results in a significant increase in brain glucose responses, possibly due to normalisation of cerebral glucose transport capacity. Furthermore, individuals with a shorter duration of diabetes and greater reduction in HbA_1c_ showed greater improvement in brain glucose levels after the intervention. Additional studies are needed to identify additional factors, or better characterise factors, that can contribute to the reversibility of CNS changes in type 2 diabetes, and to better understand the implication of these findings on neurocognitive functions.

## Data Availability

The dataset generated and analysed during the current study are available from the corresponding author on reasonable request.

## Abbreviations

BBB: Blood–brain barrier
CGM: Continuous glucose monitoring
CMRglc: Rate of glucose metabolism
CNS: Central nervous system
DPP-4: Dipeptidyl peptidase 4
EasyGV: Easy Glucose Variability
FSL: Freestyle Libre
GIR: Glucose infusion rate
MRS: Magnetic resonance spectroscopy
NAA: *N*-acetylaspartate
RD: Registered dietitian
SGLT2: Sodium–glucose cotransporter 2
STEAM: Stimulated echo acquisition mode

## References

[1] Centers for Disease Control and Prevention (2020) National Diabetes Statistics Report, 2020. Atlanta, GA: Centers for Disease Control and Prevention, U.S. Dept of Health and Human Services https://www.cdc.gov/diabetes/data/statistics-report/index.html

[2] Fox KM, Gerber Pharmd RA, Bolinder B, Chen J, Kumar S (2006). Prevalence of inadequate glycemic control among patients with type 2 diabetes in the United Kingdom general practice research database: a series of retrospective analyses of data from 1998 through 2002. Clin Ther.

[3] Roberts RO, Geda YE, Knopman DS (2008). Association of duration and severity of diabetes mellitus with mild cognitive impairment. Arch Neurol.

[4] Rawlings AM, Sharrett AR, Schneider AL (2014). Diabetes in midlife and cognitive change over 20 years: a cohort study. Ann Intern Med.

[5] Ott A, Stolk RP, van Harskamp F (1999). Diabetes mellitus and the risk of dementia: the Rotterdam study. Neurology..

[6] Small GW, Mazziotta JC, Collins MT (1995). Apolipoprotein E type 4 allele and cerebral glucose metabolism in relatives at risk for familial Alzheimer disease. JAMA.

[7] Strachan MW, Reynolds RM, Marioni RE, Price JF (2011). Cognitive function, dementia and type 2 diabetes mellitus in the elderly. Nat Rev Endocrinol.

[8] Duelli R, Maurer MH, Staudt R (2000). Increased cerebral glucose utilization and decreased glucose transporter Glut1 during chronic hyperglycemia in rat brain. Brain Res.

[9] Takata K, Kasahara T, Kasahara M, Ezaki O, Hirano H (1990). Erythrocyte/HepG2-type glucose transporter is concentrated in cells of blood-tissue barriers. Biochem Biophys Res Commun.

[10] de Graaf RA, Pan JW, Telang F (2001). Differentiation of glucose transport in human brain gray and white matter. J Cereb Blood Flow Metab.

[11] Pardridge WM, Triguero D, Farrell CR (1990). Downregulation of blood-brain barrier glucose transporter in experimental diabetes. Diabetes..

[12] Hwang JJ, Jiang L, Hamza M et al (2017) Blunted rise in brain glucose levels during hyperglycemia in adults with obesity and T2DM. JCI Insight 2(20):e95913 https://insight.jci.org/articles/view/95913 10.1172/jci.insight.95913 PMC5846903 29046482

[13] Gruetter R, Novotny EJ, Boulware SD, Rothman DL, Shulman RG (1996). 1H NMR studies of glucose transport in the human brain. J Cereb Blood Flow Metab.

[14] Gruetter R, Rothman DL, Novotny EJ (1992). Detection and assignment of the glucose signal in 1H NMR difference spectra of the human brain. Magn Reson Med.

[15] Thompson RB, Allen PS (2001). Response of metabolites with coupled spins to the STEAM sequence. Magn Reson Med.

[16] Provencher SW (1993). Estimation of metabolite concentrations from localized in vivo proton NMR spectra. Magn Reson Med.

[17] Tkac I, Starcuk Z, Choi IY, Gruetter R (1999). In vivo 1H NMR spectroscopy of rat brain at 1 ms echo time. Magn Reson Med.

[18] Heikkila O, Lundbom N, Timonen M (2010). Evidence for abnormal glucose uptake or metabolism in thalamus during acute hyperglycaemia in type 1 diabetes--a 1H MRS study. Metab Brain Dis.

[19] Seaquist ER, Tkac I, Damberg G, Thomas W, Gruetter R (2005). Brain glucose concentrations in poorly controlled diabetes mellitus as measured by high-field magnetic resonance spectroscopy. Metab Clin Exp.

[20] Ulrich EL, Akutsu H, Jurgen F et al (2008) D-(+)-glucose (C6H12O6). Biological Magnetic Resonance Data Bank. Nucleic Acids Res 36:D402–D408. 10.1093/nar/gkm957 Available from: https://bmrb.io/metabolomics/mol_summary/show_data.php?id=bmse000015. Accessed 12 April 2020

[21] Tal A, Kirov II, Grossman RI, Gonen O (2012). The role of gray and white matter segmentation in quantitative proton MR spectroscopic imaging. NMR Biomed.

[22] Hill NR, Oliver NS, Choudhary P (2011). Normal reference range for mean tissue glucose and glycemic variability derived from continuous glucose monitoring for subjects without diabetes in different ethnic groups. Diabetes Technol Ther.

[23] Chan CL, Pyle L, Newnes L (2015). Continuous glucose monitoring and its relationship to hemoglobin A1c and oral glucose tolerance testing in obese and prediabetic youth. J Clin Endocrinol Metab.

[24] Hwang JJ, Jiang L, Sanchez Rangel E (2019). Glycemic variability and brain glucose levels in type 1 diabetes. Diabetes..

[25] American Diabetes Association (2018). 4. Lifestyle management: standards of medical Care in Diabetes-2018. Diabetes Care.

[26] Hwang JJ, Jiang L, Hamza M (2017). The human brain produces fructose from glucose. JCI Insight.

[27] Gruetter R, Ugurbil K, Seaquist ER (1998). Steady-state cerebral glucose concentrations and transport in the human brain. J Neurochem.

[28] Gutniak M, Blomqvist G, Widen L, et al. D-[U-11C]glucose uptake and metabolism in the brain of insulin-dependent diabetic subjects. Am J Phys 1990;258(5 Pt 1):E805–E812. 10.1152/ajpendo.1990.258.5.E805 10.1152/ajpendo.1990.258.5.E805 2185663

[29] Boyle PJ, Kempers SF, O'Connor AM, Nagy RJ (1995). Brain glucose uptake and unawareness of hypoglycemia in patients with insulin-dependent diabetes mellitus. N Engl J Med.

[30] Henry PG, Criego AB, Kumar A, Seaquist ER (2010). Measurement of cerebral oxidative glucose consumption in patients with type 1 diabetes mellitus and hypoglycemia unawareness using (13)C nuclear magnetic resonance spectroscopy. Metab Clin Exp.

[31] Andersen JV, Christensen SK, Nissen JD, Waagepetersen HS (2017). Improved cerebral energetics and ketone body metabolism in db/db mice. J Cereb Blood Flow Metab.

[32] Pelligrino DA, LaManna JC, Duckrow RB, Bryan RM, Harik SI (1992). Hyperglycemia and blood-brain barrier glucose transport. J Cereb Blood Flow Metab.

[33] Makimattila S, Malmberg-Ceder K, Hakkinen AM (2004). Brain metabolic alterations in patients with type 1 diabetes-hyperglycemia-induced injury. J Cereb Blood Flow Metab.

[34] Brownlee M (2005). The pathobiology of diabetic complications: a unifying mechanism. Diabetes..

[35] Simpson IA, Appel NM, Hokari M (1999). Blood-brain barrier glucose transporter: effects of hypo- and hyperglycemia revisited. J Neurochem.

[36] Kumagai AK, Kang YS, Boado RJ, Pardridge WM (1995). Upregulation of blood-brain barrier GLUT1 glucose transporter protein and mRNA in experimental chronic hypoglycemia. Diabetes..

[37] Wang WT, Lee P, Yeh HW, Smirnova IV, Choi IY (2012). Effects of acute and chronic hyperglycemia on the neurochemical profiles in the rat brain with streptozotocin-induced diabetes detected using in vivo (1)H MR spectroscopy at 9.4 T. J Neurochem.

[38] Criego AB, Tkac I, Kumar A (2005). Brain glucose concentrations in healthy humans subjected to recurrent hypoglycemia. J Neurosci Res.

[39] Criego AB, Tkac I, Kumar A (2005). Brain glucose concentrations in patients with type 1 diabetes and hypoglycemia unawareness. J Neurosci Res.

[40] Frank S, Heinze JM, Fritsche A (2016). Neuronal food reward activity in patients with type 2 diabetes with improved glycemic control after bariatric surgery. Diabetes Care.

[41] King P, Peacock I, Donnelly R (1999). The UK prospective diabetes study (UKPDS): clinical and therapeutic implications for type 2 diabetes. Br J Clin Pharmacol.

[42] Nathan DM, Genuth S, The Diabetes Control and Complications Trial Research Group (1993). The effect of intensive treatment of diabetes on the development and progression of long-term complications in insulin-dependent diabetes mellitus. N Engl J Med.

[43] Lam TK, Gutierrez-Juarez R, Pocai A, Rossetti L (2005). Regulation of blood glucose by hypothalamic pyruvate metabolism. Science..

[44] Osundiji MA, Lam DD, Shaw J (2012). Brain glucose sensors play a significant role in the regulation of pancreatic glucose-stimulated insulin secretion. Diabetes..

[45] Reno CM, Puente EC, Sheng Z (2017). Brain GLUT4 knockout mice have impaired glucose tolerance, decreased insulin sensitivity, and impaired hypoglycemic Counterregulation. Diabetes..

[46] Yang R, Pedersen NL, Bao C (2019). Type 2 diabetes in midlife and risk of cerebrovascular disease in late life: a prospective nested case-control study in a nationwide Swedish twin cohort. Diabetologia..

[47] Crane PK, Walker R, Hubbard RA (2013). Glucose levels and risk of dementia. N Engl J Med.

[48] Turner CR, Holman RR, Cull CA (1998). Intensive blood-glucose control with sulphonylureas or insulin compared with conventional treatment and risk of complications in patients with type 2 diabetes (UKPDS 33). UK prospective diabetes study (UKPDS) group. Lancet..

[49] Patel A, MacMahon S, The Advance Collaborative Group (2008). Intensive blood glucose control and vascular outcomes in patients with type 2 diabetes. N Engl J Med.

[50] Geijselaers SLC, Sep SJS, Stehouwer CDA, Biessels GJ (2015). Glucose regulation, cognition, and brain MRI in type 2 diabetes: a systematic review. Lancet Diabetes Endocrinol.

[51] Launer LJ, Miller ME, Williamson JD (2011). Effects of intensive glucose lowering on brain structure and function in people with type 2 diabetes (ACCORD MIND): a randomised open-label substudy. Lancet Neurol.

[52] Zimering MB, Knight J, Ge L, Bahn G, Investigators V (2016). Predictors of cognitive decline in older adult type 2 diabetes from the veterans affairs diabetes trial. Front Endocrinol (Lausanne).

[53] Kleinridders A, Ferris HA, Reyzer ML (2018). Regional differences in brain glucose metabolism determined by imaging mass spectrometry. Mol Metab.

